# Relationship Between Fish Oil Use and Incidence of Primary Liver Cancer: Findings From a Population-Based Prospective Cohort Study

**DOI:** 10.3389/fnut.2021.771984

**Published:** 2021-12-31

**Authors:** Wei Jiang, Fu-Rong Li, Huan-Huan Yang, Guo-Chong Chen, Yong-Fei Hua

**Affiliations:** ^1^Department of Hepatopancreatobiliary Surgery, Ningbo Medical Center Lihuili Hospital, Ningbo University, Ningbo, China; ^2^School of Medicine, Southern University of Science and Technology, Shenzhen, China; ^3^Department of Epidemiology, School of Public Health, Southern Medical University, Guangzhou, China; ^4^Wanke School of Public Health, Tsinghua University, Beijing, China; ^5^Department of Epidemiology and Population Health, Albert Einstein College of Medicine, Bronx, NY, United States; ^6^Department of Nutrition and Food Hygiene, School of Public Health, Soochow University, Suzhou, China

**Keywords:** fish, fish oil, hepatocellular carcinoma, liver cancer, polyunsaturated fatty acids

## Abstract

**Background:** N-3 long-chain polyunsaturated fatty acids (LCPUFAs) prevented non-alcoholic steatohepatitis (NASH) and hepatocellular carcinoma (HCC) in studies of mouse models. We examined prospective relationships between fish oil use and risk of primary liver cancer and the major histological subtypes, such as HCC and intrahepatic cholangiocarcinoma (ICC).

**Methods:** We included 434,584 middle-aged and older men and women who were free of cancer at recruitment of the UK Biobank (2006–2010). Information on fish oil use and other dietary habits was collected via questionnaires. Cox proportional hazards models were used to compute the hazard ratio (HR) and 95% CI of liver cancer associated with fish oil use, with adjustment for socio-demographic, lifestyle, dietary, and other clinical risk factors.

**Results:** At baseline, 31.4% of participants reported regular use of fish oil supplements. During a median of 7.8 years of follow-up, 262 incident liver cancer cases were identified, among which 127 were HCC and 110 were ICC cases. As compared with non-users, fish oil users had a significantly 44% (95% CI: 25–59%) lower risk of total liver cancer, and 52% (95% CI: 24–70%) and 40% (95% CI: 7–61%) lower risk of HCC and ICC, respectively. Higher intake of oily fish also was associated with a lower risk of HCC (≥2 vs. <1 serving/week: HR = 0.46; 95% CI: 0.23–0.96; *P*-trend = 0.027) but not ICC (*P*-trend = 0.96).

**Conclusion:** Habitual use of fish oil supplements was associated lower risk of primary liver cancer regardless of cancer histological subtypes, potentially supporting a beneficial role of dietary n-3 LCPUFAs in liver cancer prevention.

## Introduction

In studies of mouse models, marine-derived n-3 long-chain polyunsaturated fatty acids (LCPUFAs), such as eicosapentaenoic acid (EPA) and docosahexaenoic acid (DHA), prevented Western diet-induced non-alcoholic steatohepatitis (NASH) (1, 2) and decreased the risk of carcinogen-induced hepatocellular carcinoma (HCC) (3). In human feeding studies of patients with chronic liver disease (e.g., non-alcoholic fatty liver disease [NAFLD] or NASH), supplementation with fish oil (a mix of EPA and DHA) reduced liver fat content and favorably modified the lipidomic profile underlying hepatic dysfunction (4, 5). These biological actions associated with fish oil supplements indicate a possible role of dietary n-3 LCPUFA in liver cancer prevention (6).

A few prospective observational studies of the human population have suggested that higher fish (7–9) or dietary n-3 LCPUFA intake (9) was associated with a lower risk of HCC. Two previous meta-analyses of the published case-control and cohort studies have suggested moderate inverse associations between total fish or dietary n-3 LCPUFA intake and the risk of liver cancer. However, a more recent umbrella review evaluated both meta-analyses and concluded that current epidemiologic evidence for a protective effect of dietary n-3 LCPUFA intake on liver cancer was “weak,” which highlights the need for additional data from large population studies. Moreover, previous studies have been focused on dietary n-3 LCPUFA intake from food sources (mainly fish) in relation to the risk of liver cancer, and the findings may be confounded by the contamination of fish (10), fish preparation method (11, 12), or other nutrients, such as protein, vitamins, and minerals in fish. To the best of our knowledge, no previous studies have evaluated the relationship between fish oil supplements and risk of liver cancer. An additional open question is how n-3 LCPUFA intake may be associated with the risk of intrahepatic cholangiocarcinoma (ICC), another major histological subtype of liver cancer beyond HCC.

Using data from a large population-based cohort study (UK Biobank), we examined prospective relationships between habitual use of fish oil supplements and risk of primary liver cancer and its major histologic subtypes, such as HCC and ICC. We also examined the consumption of fish defined by fat content (i.e., oily and non-oily) and risk of these cancers.

## Methods

### Study Population

The UK Biobank is a large prospective cohort study established to provide a resource for the investigation of the genetic, environmental, and lifestyle factors associated with a wide range of diseases, namely, cancer (13). Between 2006 and 2010, ~500,000 ethnically diverse men and women aged 37–73 years were recruited from 22 centers located across England, Wales, and Scotland. At recruitment, participants provided a wide range of information on health and diseases and underwent various measurements. The UK Biobank received ethical approval from the research ethics committee (REC reference for UK Biobank 11/NW/0382) and at recruitment, all participants provided written informed consent.

### Assessments of Fish Oil Use and Habitual Diet

As described elsewhere (14), information on habitual use of fish oil supplements in the UK Biobank was collected at recruitment via a touchscreen questionnaire by asking participants: “Do you regularly take any of the following?”. In addition, participants were also invited to report their status of using fish oil (over the past 24 h) during 5 rounds of 24-h dietary recalls conducted between 2009 and 2012, and these data were used in our sensitivity analysis described below.

Information on usual dietary factors was collected via a touchscreen food-frequency questionnaire that included 29 questions concerning the average intake of major foods or food groups over the past year. The questions on consumption of oily fish, non-oily fish, and meat had six frequency categories ranging from “never” to “once or more daily.” For fruit, vegetables, cereal, and coffee, participants were asked to directly enter the integer number of pieces, heaped tablespoons, bowls, and cups, respectively, that they ate or drank per day; or select “less than one” if the consumption was less than daily.

### Ascertainment of Cancer

The UK Biobank is linked to cancer registry data from the Health and Social Care Information Center (in England and Wales) and the National Health Service Central Register (in Scotland). Both information on self-reported physician's diagnosis and International Classification of Diseases (ICD)-10 codes were used to identify cancer patients at baseline. Incident cases of overall liver cancer, HCC, and ICC during follow-up (until December 14, 2016) were identified using ICD-10 codes C22, C22.0, and C22.1, respectively (15).

### Assessment of Other Covariates

Information on demographic and socioeconomic factors, lifestyle behaviors, reproductive and medical histories, and medication use was collected at baseline via a touchscreen questionnaire and nurse-led interviews. The Townsend deprivation index was generated to reflect socioeconomic status of the participants. Body mass index (BMI) was calculated using measured weight and height (kg/m^2^). Prevalent diabetes at baseline was ascertained through multiple procedures, such as self-report, ICD-10 code E11, and blood hemoglobin A1c, as reported previously (14). Baseline physical activity was assessed using the self-reported short-form international physical activity questionnaire, and data were summarized and reported in metabolic equivalent-hours per week. Various blood analytes, such as biomarkers of liver function (e.g., alanine aminotransferase and total bilirubin), were determined using appropriate methods (16).

### Statistical Analysis

We excluded participants who had prevalent cancer rather than non-melanoma skin cancer (*n* = 52,424), did not report information on fish oil use (*n* = 5,642), other major food groups (*n* = 9,773), or withdrew from the study (*n* = 87). As a result, 434,584 participants (203,428 men and 231,156 women) remained for the present analysis.

Baseline participant characteristics were described for the overall study population by fish oil use status. Cox proportional hazards models were used to compute the hazard ratio (HR) and 95% CI of liver cancer associated with habitual use of fish oil supplements (yes vs. no). Person-time of follow-up was calculated from the date of enrollment through the date of diagnosis of liver cancer, death or withdrawal from the study, or end of the most recent follow-up, whichever came first. Except for an age-and-sex-adjusted model, a full model was applied by further adjusting for ethnic group, Townsend deprivation index, smoking status, pack-years of smoking (for current smokers), alcohol consumption, total physical activity, dietary factors (cereal, fresh fruit, fresh vegetables, red meat, processed meat, oily fish, and non-oily fish), BMI, diabetes, and blood measures of liver function (*z* scores of alanine aminotransferase and total bilirubin).

We performed stratified analyses and tested for potential interactions of fish oil use with age, sex, smoking status, alcohol consumption, BMI, and diabetes status at baseline. Several sensitivity analyses were performed to test the robustness of the findings by (1) excluding participants (*n* = 1,315) who had chronic viral hepatitis (ICD-10 codes B18), alcoholic liver disease (ICD-10 codes K70), or liver fibrosis/cirrhosis (ICD-10 codes K70) at baseline; (2) excluding participants (*n* = 14,650) who died from any causes other than liver cancer during follow-up to examine the potential impact of competing risk; (3) excluding incident cases of liver cancer within the first 3 (*n* = 89) or 5 (*n* = 156) years of follow-up to address potential reverse causation; and (4) defining fish oil users as participants who reported use of fish oil both at baseline and during at least one of the 24-h dietary recalls and non-users as those who neither reported use at baseline nor during 24-h dietary recalls. This analysis was restricted to participants (*n* = 187,120) with at least one 24-h dietary recall and excluded those (*n* = 27,334) who reported fish oil use at baseline or 24-h dietary recalls only (14).

Finally, we examined associations of oily and non-oily fish intakes with the risk of liver cancer, HCC, and ICC. Four intake categories (never, <1, 1, and ≥2 servings/week) were created for both intakes by combining the upper categories to allow for the meaningful number of cases in each category. Statistical analyses were performed using Stata (version 15.1; StataCorp, College Station, TX, USA).

## Results

### Participant Characteristics

At baseline, 31.4% (*n* = 136,252) of participants reported regular use of fish oil supplements. Participants who reported regular use of fish oil supplements, as compared with non-users, were older, were more likely to be women and alcohol drinkers, had a higher level of physical activity, and were less likely to be current smokers, be obese, or have diabetes (Table 1). Fish oil users also tended to have a healthier eating pattern characterized by higher intakes of fresh fruit and vegetables and lower processed meat intake.

**Table 1:** Baseline participant characteristics according to fish oil use.

	**Overall**	**Habitual fish oil use**	
		**No**	**Yes**
No of participants	434,584	298,332	136,252
Age, y	56.3 ± 8.1	55.3 ± 8.2	58.4 ± 7.5
Men, %	46.8	47.9	44.4
British white, %	88.8	88.5	89.5
Townsend deprivation index >0^a^, %	28.2	29.4	25.4
**Smoking status, %**			
Never	55.4	55.8	54.3
Former	34.3	32.8	37.6
Current	10.2	11.4	8.1
**Alcohol drinking, %**			
Never	4.2	4.4	3.7
Former	3.5	3.6	3.3
Current <1 drink/week	22.4	22.7	21.7
Current 1–2 drinks/week	25.9	25.9	26.0
Current ≥3 drinks/week	44.0	43.4	45.3
Total physical activity, MET-h/week	44.3 ± 45.2	42.7 ± 44.7	47.8 ± 46.2
**Body mass index**			
<25 kg/m^2^	33.1	32.5	34.2
25– <30 kg/m^2^	42.7	42.2	43.7
≥30 kg/m^2^	24.3	25.3	22.1
Diabetes, %	6.0	6.3	5.5
**Dietary factors**			
Coffee, cups/d	2.0 ± 2.1	2.1 ± 2.2	1.9 ± 1.9
Cereal, bowls/d	0.6 ± 0.4	0.6 ± 0.4	0.7 ± 0.4
Fresh fruit, pieces/d	2.2 ± 1.6	2.1 ± 1.6	2.5 ± 1.6
Fresh vegetables, heaped tablespoons/d	4.9 ± 3.4	4.8 ± 3.4	5.1 ± 3.3
Red meat ≥2 servings/week, %	50.3	50.5	49.9
Processed meat ≥2 servings/week, %	31.3	32.5	28.5
Oily fish ≥2 servings/ week, %	17.9	15.8	22.5
Non-oily fish ≥2 servings/week, %	16.4	15.6	18.1
**Liver function**			
Alanine aminotransferase, median (IQR) U/L	20.1 (15.7–26.9)	20.2 (15.3–27.7)	20.3 (15.8–27.2)
Total bilirubin, median (IQR) umol/L	8.1 (6.6–10.3)	8.1 (6.5–10.5)	8.1 (6.4–10.4)

*Data are presented as mean ± SD unless where otherwise indicated*.

*IQR, interquartile range; MET, metabolic equivalent*.

a *A higher Townsend deprivation index indicates a greater degree of deprivation (or lower socioeconomic status)*.

### Fish Oil Use and Risk of Liver Cancer

During a median of 7.8 years of follow-up (3,379,073 person-years), 262 incident cases of liver cancer were documented, among which 127 were HCC and 110 were ICC cases. After age and sex adjustment, there were significant inverse associations between fish oil use and risk of total liver cancer and its major histological subtypes, such as HCC and ICC. These inverse associations were only slightly attenuated after further adjustment for socio-demographic, lifestyle, dietary, and other clinical risk factors (Table 2). The fully adjusted HR of total liver cancer was 0.56 (95% CI: 0.41–0.75; *P* = 0.0001) when comparing fish oil users with non-users, with corresponding HRs of 0.48 (95% CI: 0.30–0.76; *P* = 0.0019) for HCC and 0.60 (95% CI: 0.39–0.93; *P* = 0.022) for ICC.

**Table 2:** Association of fish oil use with the risk of liver cancer and its major histological subtypes.

	**Habitual fish oil use**		
	**No (*n* = 298,332)**	**Yes (*n* = 136,252)**	***P*-value**
**Liver cancer**			
No. of cases	205	57	
No. of person-years	2,316,771	1,062,302	
Age and sex-adjusted [HR (95% CI)]	1.00 (Referent)	0.50 (0.37–0.67)	<0.0001
Multivariable adjusted [HR (95% CI)]	1.00 (Referent)	0.56 (0.41–0.75)	0.0001
**Hepatocellular carcinoma**			
No. of cases	105	22	
No. of person-years	2,316,892	1,062,348	
Age and sex-adjusted [HR (95% CI)]	1.00 (Referent)	0.38 (0.24–0.61)	0.0001
Multivariable adjusted [HR (95% CI)]	1.00 (Referent)	0.48 (0.30–0.76)	0.0019
**Intrahepatic cholangiocarcinoma**			
No. of cases	82	28	
No. of person-years	2,316,955	1,062,336	
Age and sex-adjusted [HR (95% CI)]	1.00 (Referent)	0.59 (0.38–0.91)	0.018
Multivariable adjusted [HR (95% CI)]	1.00 (Referent)	0.60 (0.39–0.93)	0.022

*Multivariable models were adjusted for age (y), sex, ethnic group (White, other), Townsend deprivation index, smoking status (never, former, current), pack-years of smoking (for current smokers), alcohol consumption [never, former, current (<1, 1–2, ≥3 drinks/week)], total physical activity (MET-h/week), dietary factors (consumption of cereal, fresh fruit, fresh vegetables, red meat, processed meat, oily fish, non-oily fish, and coffee), body mass index (kg/m^2^), diabetes, and blood measures of liver function (z scores of alanine aminotransferase and total bilirubin)*.

### Subgroup and Sensitivity Analyses

**Figure 1:**
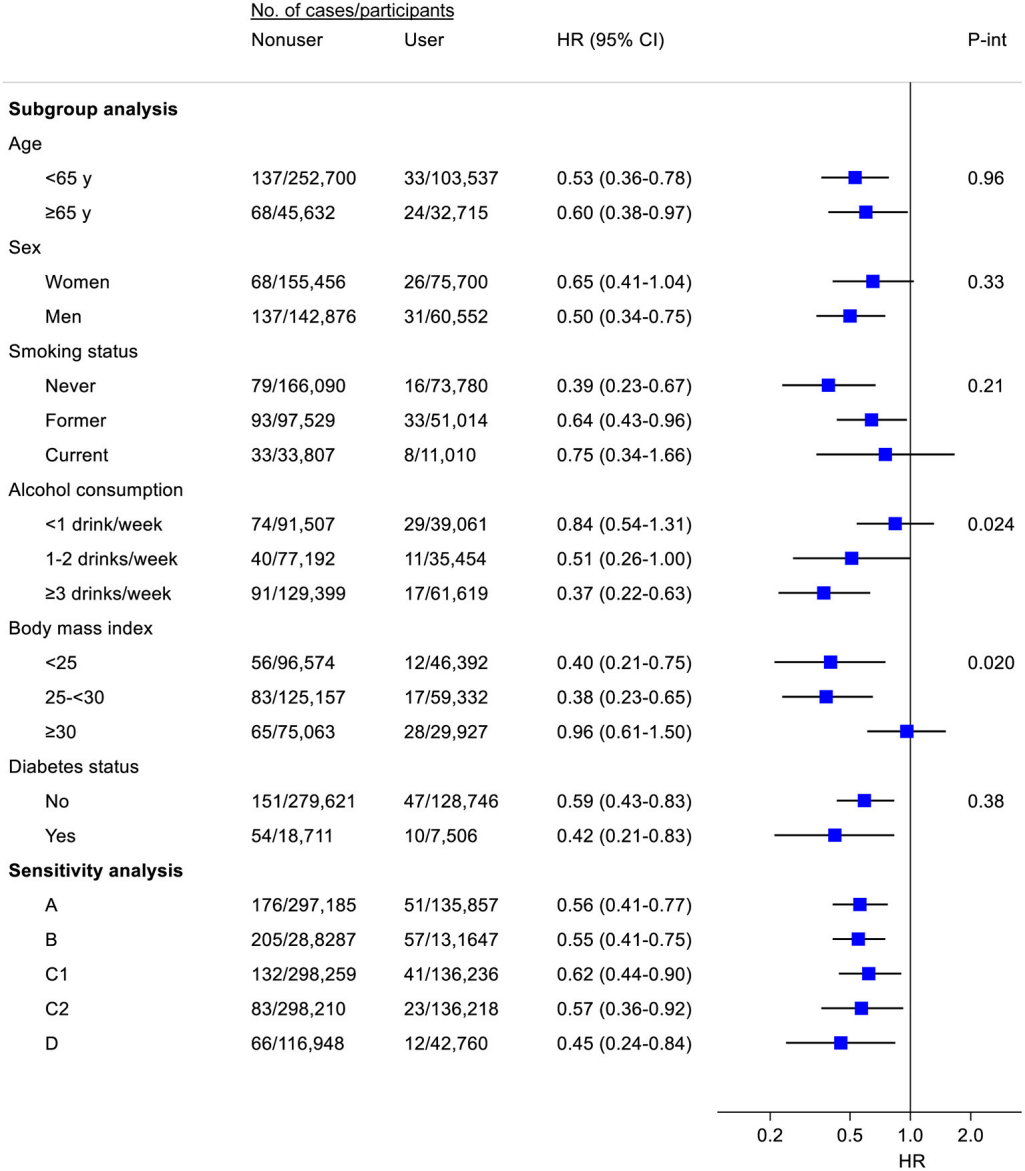
Subgroup and sensitivity analyses for the association of fish oil use with the risk of liver cancer. Where appropriate, results were adjusted for age (y), sex, ethnic group (White, other), Townsend deprivation index, smoking status (never, former, current), pack-years of smoking (for current smokers), alcohol consumption [never, former, current (<1, 1–2, ≥3 drinks/week)], total physical activity (MET-h/week), dietary factors (consumption of cereal, fresh fruit, fresh vegetables, red meat, processed meat, oily fish, non-oily fish, and coffee), body mass index (kg/m^2^), diabetes, and blood measures of liver function (*z* scores of alanine aminotransferase and total bilirubin). A, Excluding participants with viral hepatitis, alcoholic liver disease, or liver fibrosis/cirrhosis; B, Excluding non-liver cancer deaths during follow-up; C, Excluding incident cases of liver cancer within the first 3 (C1) or 5 (C2) years of follow-up; D, Among participants (*n* = 187,120) with ≥1 24-h dietary recall, risk of liver cancer for participants who reported fish oil use both at baseline and at ≥1 of 24-h dietary recalls vs. risk for participants who neither reported fish use at baseline nor during 24-h dietary recalls. Participants (*n* = 27,334) who reported fish oil use at baseline or 24-h dietary recalls only were excluded from this analysis.

The inverse association of fish oil use with the risk of total liver cancer did not vary by baseline age, sex, smoking status, or diabetes of the participants (Figure 1). However, the association appeared to be stronger among regular (1–2 drinks/week) or heavier drinkers (1–2 or ≥3 drinks/week) than among light drinkers or non-drinkers (<1 drink/week) (*P* for interaction = 0.024). Stratifying by BMI, the inverse association was observed in lean and overweight participants, but not among those who were obese (*P* for interaction = 0.020). Results were stable in several pre-defined sensitivity analyses (e.g., excluding participants with viral hepatitis, alcoholic liver disease, or liver fibrosis/cirrhosis at baseline). Notably, in a subsample (*n* = 187,120) where at least one 24-h dietary recall was conducted, participants who reported fish oil use both at baseline and at any of the 24-h dietary recalls had 55% (HR = 0.45, 95% CI: 0.24–0.84) lower risk of liver cancer when compared with those who neither reported fish use at baseline nor during 24-h dietary recalls.

### Fish Intake and Risk of Liver Cancer

There was a suggestive, non-significant association between higher oily fish intake and lower risk of total liver cancer (≥2 vs. <1 serving/week: HR = 0.68; 95% CI 0.41–1.13; *P*-trend = 0.087) (Table 3). Oily fish intake was significantly and inversely associated with the risk of HCC (≥2 vs. <1 serving/week: HR = 0.46; 95% CI 0.23–0.96; *P*-trend = 0.027) but not ICC (*P*-trend = 0.96). No association was found for non-oily fish and risk of liver cancer or its histological subtypes.

**Table 3:** Association of fish intake with risk of liver cancer and its major histological subtypes.

	**Oily fish**		**Non-oily fish**	
	**No. of cases/ participants**	**HR (95% CI)**	**No. of cases/ participants**	**HR (95% CI)**
**Liver cancer**				
Never	38/47,713	1.00 (Referent)	16/20,372	1.00 (Referent)
<1 serving/ week	87/144,988	0.87 (0.57–1.35)	75/126,445	0.85 (0.46–1.57)
1 serving/week	93/164,110	0.77 (0.49–1.20)	129/216,732	0.88 (0.48–1.61)
≥2 servings/ week	44/77,773	0.68 (0.41–1.13)	42/71,035	0.87 (0.45–1.69)
P-trend		0.087		0.83
**Hepatocellular carcinoma**				
Never	22/47,713	1.00 (Referent)	8/20,372	1.00 (Referent)
<1 serving/ week	41/144,988	0.75 (0.42–1.37)	39/126,445	1.01 (0.43–2.38)
1 serving/week	46/164,110	0.70 (0.38–1.28)	58/216,732	0.98 (0.41–2.30)
≥2 servings/ week	18/77,773	0.46 (0.23–0.96)	22/71,035	1.12 (0.44–2.82)
P-trend		0.027		0.84
**Intrahepatic cholangiocarcinoma**				
Never	13/47,713	1.00 (Referent)	7/20,372	1.00 (Referent)
<1 serving/ week	35/144,988	1.00 (0.48–2.09)	27/126,445	0.61 (0.23-1.61)
1 serving/week	39/164,110	0.88 (0.41–1.85)	60/216,732	0.76 (0.29–1.95)
≥2 servings/ week	23/77,773	1.06 (0.47–2.36)	16/71,035	0.62 (0.22–1.74)
P-trend		0.96		0.71

*Results were adjusted for age (y), sex, ethnic group (White, other), Townsend deprivation index, smoking status (never, former, current), pack-years of smoking (for current smokers), alcohol consumption [never, former, current (<1, 1–2, ≥3 drinks/week)], total physical activity (MET-h/week), dietary factors (consumption of cereal, fresh fruit, fresh vegetables, red meat, processed meat, oily fish or non-oily fish for each other, and coffee), body mass index (kg/m^2^), diabetes, blood measures of liver function (z scores of alanine aminotransferase and total bilirubin), and fish oil use*.

## Discussion

In this prospective analysis of UK men and women with more than 3.3 million person-years of follow-up, habitual use of fish oil supplements was associated with a lower risk of liver cancer regardless of cancer histological subtypes (i.e., HCC and ICC). As compared with fish oil non-users, participants who reported regular use of fish oil supplements had a 44% lower risk of total liver cancer, and 52 and 40% lower risk of HCC and ICC, respectively. Higher intake of oily fish also was associated with a lower risk of HCC, while no association was found between non-oily fish intake and risk of liver cancer or its subtypes.

To our knowledge, this is the first study of fish oil use and the risk of liver cancer or its histologic subtypes. Several prospective studies have assessed the relationships between total fish or dietary n-3 LCPUFA intake and risk of liver cancer (mostly HCC). In a meta-analysis that summarized data from five cohort studies published before 2014, a higher intake of total fish was associated with a significantly 18% lower risk of liver cancer. This is in line with more recent findings from two large prospective cohorts of US health professionals in which total fish consumption was inversely associated with the risk of HCC (8). While the majority of these studies did not take into account the fat content of fish, two prospective studies of Asian populations have examined dietary n-3 LCPUFA intake in relation to the risk of HCC. In a cohort of 90,296 Japanese men and women, both higher intakes of n-3 LCPUFA-rich fish and individual n-3 LCPUFA (i.e., EPA and DHA) were associated with a lower risk of HCC. In another cohort of Singapore Chinese, however, n-3 LCPUFA intake (above vs. below median) was not associated with the risk of HCC. The potential reasons for the study-specific differences in the association are unclear. Possibly, they might be partially attributable to geographic variation in the degree of contamination of fish (e.g., persistent organic pollutants) (10) or population differences in fish preparation method that may affect the fatty acid composition and energy density of fish consumed (11, 12). Our analysis focused on the association for fish oil supplements and is less influenced by these potential confounders or by other nutrients in fish.

In subgroup analysis, we observed that the lower risk of liver cancer associated with fish oil use was limited to alcohol drinkers and non-obese participants. While the possibility of chance findings cannot be excluded, there is biological plausibility underlying such potential effect modifications. In studies of the human population, wine drinking has been associated with increased levels of n-3 LCPUFA both in plasma and in red blood cells, independent of dietary n-3 LCPUFA intake (17, 18). This is further evidenced by findings from a rat model study, in which 7 weeks of moderate drinking resulted in elevated plasma n-3 LCPUFA levels (+65% for EPA and +19% for DHA) (19). Such an interaction was referred to as the “fish-like effect of moderate drinking” (17) and may involve activation of the elongation/desaturation pathway following alcohol drinking, thereby increased synthesis of n-3 LCPUFA from the precursor α-linolenic acid (18). On the other hand, the metabolisms of n-3 LCPUFA may be reduced by obesity (e.g., due to obesity-induced oxidative injury to erythrocyte membranes) (20, 21).

N-3 LCPUFA is involved in a wide range of molecular and cellular pathways. For example, n-3 LCPUFA can alter the physical and chemical properties of cell membranes and modulate membrane channels and proteins, and they also serve as key constituents of the circulating lipid pools and convert to bioactive metabolites involved in signal transduction (22–25). With respect to liver cancer, experimental evidence indicates that n-3 LCPUFA could inhibit the growth of hepatobiliary tumor cells, partially through blocking beta-catenin and cyclooxygenase-2 signaling pathways (26, 27). As an advanced form of NFALD, NASH can progress to cirrhosis, which is a strong risk factor for liver cancer. In studies of mouse models, n-3 LCPUFA prevented Western diet-induced NASH, possibly through suppression of major phenotypic features of NASH, such as hepatic inflammation, oxidative stress, steatosis, and fibrosis (1, 2, 28). In mice fed a high-fat diet, supplementation with EPA prevented the development of carcinogen-induced HCC (3). In human feeding studies of patients with NAFLD or NASH, supplementation with fish oil (EPA + DHA) reduced liver fat content and favorably modified the lipidomic profile underlying hepatic dysfunction (4, 5).

In the UK Biobank, information on the duration of use, dose, or formulation (relative percentage of EPA and DHA) of fish oil was not available. Animal studies have suggested that DHA may be more effective than EPA in the suppression of diet-induced steatohepatitis (1, 2), although both fatty acid intakes have been associated with a lower risk of HCC in the human population (9). Data from randomized controlled trials in humans also indicated that DHA may be more effective than EPA in modulating specific disease risk factors, such as inflammation markers (29). EPA and DHA levels present in different biological moieties (e.g., plasma, cellular membranes, and adipose tissue) are good biomarkers for dietary n-3 LCPUFA intake (30). Omega-3 Index (O3I), which is the proportion of EPA and DHA in red blood cell membranes, could be a marker for dietary n-3 LCPUFA intake in the past months due to the relatively long half-life of erythrocytes (~120 days) (30). O3I could be significantly increased by fish oil supplementation (31–33), and it also serves as an indicator of disease onset/progression given that increased contents of monounsaturated fatty acids, altered levels of EPA and DHA/ALA (α-linolenic acid) ratio and, in general, PUFA imbalances have been associated with increased risk of various diseases in epidemiologic studies, such as cancer (25, 34–36). How O3I or other indicators of the bioavailability may be influenced by fish oil supplementation with different fatty acid components, doses, or durations and the corresponding clinical significance needs to be explored further.

Strengths of our study include its prospective and population-based design, the assessment of both HCC and ICC that have not been evaluated for the association with dietary n-3 PUFA before, the adjustment for various important clinical risk factors, and the stable results observed in different sensitivity analyses addressing possible study bias. Apart from the abovementioned limitations, several additional limitations should be noted when interpreting our findings. First, causal inference cannot be made from this observational study. Second, our analysis mostly used baseline fish oil data and longitudinal changes in fish oil use may have attenuated the examined associations due to potential regression dilution bias. In the analysis where fish oil users were defined as participants who reported use of fish oil at multiple interviews, as expected, the association of fish oil use with a risk of liver cancer became slightly stronger. Third, antigens for hepatitis B or C virus were measured only for a small proportion of participants (<2%) in the UK Biobank, and thus further interaction analyses are not feasible. However, dietary n-3 LCPUFA intake has been associated with a lower risk of HCC irrespective of hepatitis B or C infection status (7, 9). Moreover, we observed similar results after excluding clinically identified viral hepatitis, alcoholic liver disease, or liver fibrosis/cirrhosis at baseline. Finally, participants in the UK Biobank were predominantly of European descent and they may also be motivated individuals who want to participate in research and may not reflect the general population. Thus, caution is needed when generalizing our findings to other populations.

In summary, in a large population-based prospective study of UK men and women, habitual use of fish oil supplements reported at baseline was associated lower risk of liver cancer and its major histological subtypes including HCC and ICC after ~8 years. We also confirmed an inverse association between dietary oily fish intake and the risk of HCC. Additional studies of fish oil supplements, such as randomized controlled trials conducted among high-risk patients (e.g., liver fibrosis/cirrhosis), may help elucidate the clinical relevance of fish oil supplements in the treatments of risk factors and the primary prevention of liver cancer.

## Data Availability Statement

The original contributions presented in the study are included in the article/supplementary material, further inquiries can be directed to the corresponding author/s.

## Ethics Statement

The studies involving human participants were reviewed and approved by NHS National Research Ethics Service North West. The patients/participants provided their written informed consent to participate in this study.

## Author Contributions

F-RL and G-CC: study concept, design, and acquisition of data. WJ, F-RL, and G-CC: analysis and interpretation of data. WJ and G-CC: drafting of the manuscript. H-HY: visualization and validation. Y-FH and G-CC: study supervision. All authors: critical revision of the manuscript for important intellectual content.

## Conflict of Interest

The authors declare that the research was conducted in the absence of any commercial or financial relationships that could be construed as a potential conflict of interest.

## Publisher's Note

All claims expressed in this article are solely those of the authors and do not necessarily represent those of their affiliated organizations, or those of the publisher, the editors and the reviewers. Any product that may be evaluated in this article, or claim that may be made by its manufacturer, is not guaranteed or endorsed by the publisher.

## Abbreviations

CI: confidence interval
BMI: body mass index
DHA: docosahexaenoic acid
EPA: eicosapentaenoic acid
HR: hazard ratio
HCC: hepatocellular carcinoma
ICC: intrahepatic cholangiocarcinoma
ICD: International Classification of Diseases
LCPUFA: long-chain polyunsaturated fatty acids
NAFLD: non-alcoholic fatty liver disease
NASH: non-alcoholic steatohepatitis.
